# Acute pancreatitis soon after COVID-19 vaccination: A case report

**DOI:** 10.1097/MD.0000000000028471

**Published:** 2022-01-14

**Authors:** Sotaro Ozaka, Takamoto Kodera, Shimpei Ariki, Takashi Kobayashi, Kazunari Murakami

**Affiliations:** a Department of Gastroenterology, Faculty of Medicine, Oita University, Oita, Japan; b Department of Internal Medicine, Saiki Central Hospital, Oita, Japan; c Department of Infectious Disease Control, Faculty of Medicine, Oita University, Oita, Japan.

**Keywords:** acute pancreatitis, case report, COVID-19, vaccine

## Abstract

**Rationale:**

In response to the global coronavirus infectious disease 2019 (COVID-19) pandemic, several vaccines against severe acute respiratory syndrome coronavirus 2 have been developed. Although many infrequent side effects of COVID-19 mRNA vaccine have been reported, only a few cases of pancreatitis have been reported.

**Patient concerns:**

A 71-year-old woman was presented to the hospital with upper abdominal pain and vomiting. She had no history of alcohol consumption, pancreatitis, or allergic reactions to vaccines. She had received the first dose of the Pfizer/BioNTech COVID-19 mRNA vaccine 2 days prior to her current presentation. Laboratory tests revealed elevated serum pancreatic enzymes. An abdominal computed tomography scan showed diffuse enlargement of the pancreas with fat stranding extending to below the kidneys bilaterally.

**Diagnosis:**

The patient was diagnosed with acute pancreatitis.

**Interventions:**

The patient was treated with the administration of intravenous antimicrobials, proteolytic enzyme inhibitors, and proton pump inhibitors.

**Outcomes:**

The patient had an uneventful recovery with no complications.

**Lessons:**

Acute pancreatitis can develop shortly after COVID-19 mRNA vaccination. Therefore, of great importance to differentiate acute pancreatitis when abdominal pain occurs after COVID-19 mRNA vaccination.

## 1 Introduction

Several vaccines against severe acute respiratory syndrome coronavirus 2 (SARS-CoV-2) have been developed in response to the coronavirus infectious disease 2019 (COVID-19) global pandemic. These vaccines have proven to be highly effective to prevent COVID-19 in the general population.^[1–3]^ Pfizer's BNT162b2 (Pfizer/BioNTech) is a vaccine based on the SARS-CoV-2 spike-protein mRNA.^[4]^ Common side effects of this vaccine include injection site pain, transient fever, headache, and fatigue.^[1,5]^ Recently, although many infrequent side effects have also been reported, only a few cases of acute pancreatitis have been reported.^[6–8]^ Here, we report a rare case of acute pancreatitis that developed shortly after the first dose of the Pfizer/BioNTech COVID-19 mRNA vaccine (Comirnaty).

## 2 Case presentation

A 71-year-old woman with a history of hypertension, hyperlipidemia, and cerebral infarction was presented with upper abdominal pain and vomiting. She had no history of alcohol consumption, pancreatitis, or allergic reactions to vaccines. She was on regular medication with rosuvastatin, nifedipine, aspirin, enalapril, levocetirizine, and magnesium oxide, and no drug changes were made in the past 3 years. She has no family history of note.

**Figure 1:**
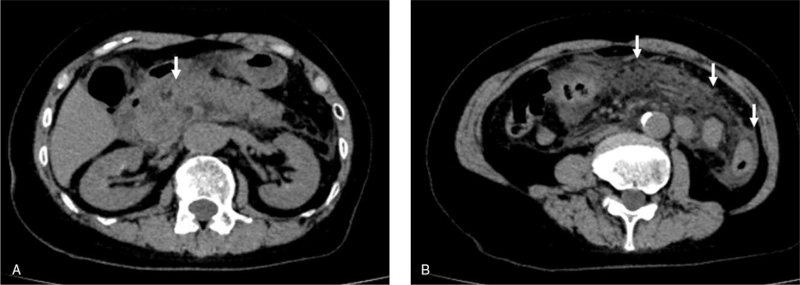
(A) Computed tomography (CT) scan demonstrating diffuse enlargement of the pancreas and ill-defined parenchymal contours (arrow). (B) CT showed fat stranding extending to below the kidneys bilaterally (arrow).

**Figure 2:**
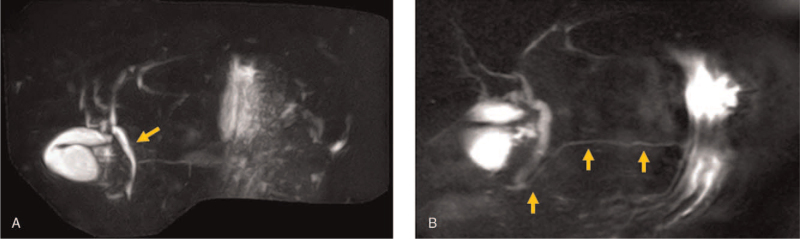
Magnetic resonance cholangiopancreatography (MRCP) showed no biliary stones or anatomical pancreaticobiliary maljunction. (A) The biliary system was normal (arrow). (B) The main pancreatic duct was not dilated (arrow). There were no cystic lesions.

**Figure 3:**
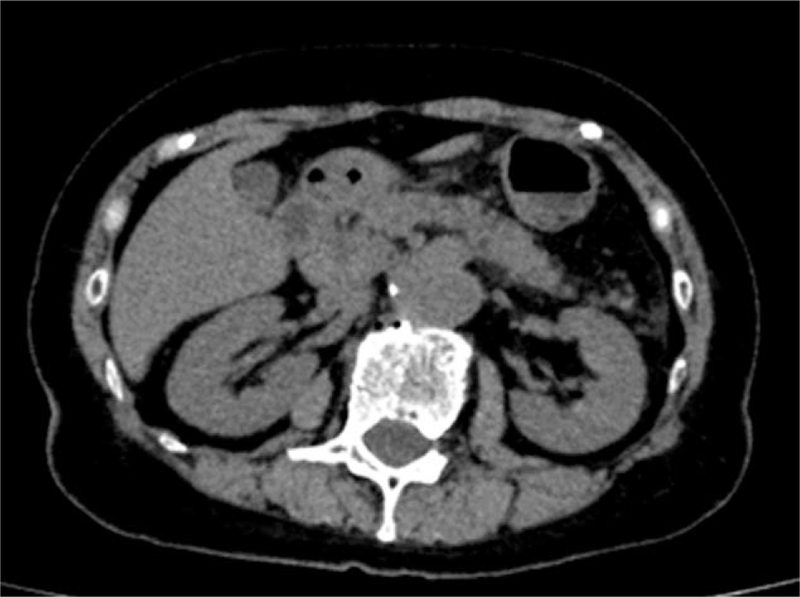
CT scan on day 14 of hospitalization revealed the improvement in the enlargement of the pancreas. CT = Computed tomography.

She had received the first dose of the Pfizer/BioNTech COVID-19 mRNA vaccine 2 days prior to her current presentation. Her blood pressure was 142/86 mmHg, heart rate was 92 bpm, and body temperature was 37.3°C. Intense tenderness was observed in the upper abdomen. Laboratory tests revealed serum amylase, 1043 IU/L (reference range, 44–132); lipase, 383 IU/L (reference range, 17–57); and elastase-1, 2158 ng/dL (reference, <300). Her serum alanine transferase, aspartate transaminase, and **γ**-glutamyl transpeptidase levels were within normal limits. Furthermore, serum triglyceride level was 55 U/L (reference range, 0–149) and immunoglobulin G4 level was 34 mg/dL (reference range, 4–108) (Table 1). An abdominal computed tomography (CT) scan showed diffuse enlargement of the pancreas with ill-defined parenchymal contours (Fig. 1A) and with fat stranding extending to below the kidneys bilaterally (Fig. 1B). Magnetic resonance cholangiopancreatography revealed no biliary stones or anatomical pancreaticobiliary maljunction (Fig. 2A, B). In addition, the abdominal ultrasound performed before CT scan showed no gallstones. According to the revised Atlanta Classification, the diagnosis of mild acute pancreatitis was made, because there were no local complications or organ failure (Modified Marshall score: 0). The patient was treated with the administration of intravenous antimicrobials, proteolytic enzyme inhibitors, and proton pump inhibitors. On day 4 of hospitalization, her serum amylase level decreased to the normal range. On day 14 of hospitalization, follow-up abdominal CT scan revealed the improvement in the enlargement of the pancreas (Fig. 3). The pancreatitis gradually improved thereafter, and she was discharged home on day 38 of hospitalization. Our patient recovered well, having no symptoms and complications after 3 months of follow-up. In addition, informed consent was obtained from the patient for publication of this case report and any accompanying images.

**Table 1:** Laboratory data on admission.

Items	Data	Reference	Items	Data	Reference	Items	Data	Reference
WBC	14,100/μL	3300–8600	Alb	4.5 g/dL	4.1–5.1	Na	136 mEq/L	138–145
RBC	493 × 10^4^/μL	386 × 10^4^–492 × 10^4^	BUN	13.1 mg/dL	8–20	Cl	100 mEq/L	101–108
Hb	14.8 g/dL	11.6–14.8	Cre	0.57 mg/dL	0.46–0.79	K	3.3 mEq/L	3.6–4.8
Hct	42.8%	35.1–44.4	LDL-C	98 mg/dL	0–139	Ca	8.9 mg/dL	8.7–11.0
MCV	86.8 fL	83.6–98.2	TG	55 U/L	0–149	AMY	1043 IU/L	44–132
MCHC	34.6%	31.7–35.3	AST	26 U/L	13–30	Lipase	383 IU/L	17–57
Plt	19.6 × 10^4^/μL	15.8 × 10^4^–34.8 × 10^4^	ALT	20 U/L	7–23	Elastase-1	2158 ng/dL	<300
			LDH	306 U/L	124–222	CRP	1.81 mg/dL	0–0.14
COVID-19 Ag	0.22 pg/mL	<3.99	γ-GTP	29 U/L	9–32	IgG4	34 mg/dL	4–108
						CA19-9	6.5 U/mL	<37

**γ**-GTP = **γ**-glutamyl transpeptidase, Alb = albumin, AST = aspartate transaminase, BUN = blood urea nitrogen, COVID-19 = coronavirus infectious disease 2019, COVID-19 Ag = coronavirus infectious disease 2019 antigen, AMY = amylase, Cre = creatinine, CRP = C-reactive protein, Hb = hemoglobin, Hct = hematocrit, IgG4 = immunoglobulin G4, LDH = lactate dehydrogenase, LDL-C = low density lipoprotein cholesterol, MCHC = mean corpuscular hemoglobin concentration, MCV = mean corpuscular volume, Plt = platelets, RBC = red blood cell, TG = triglyceride, WBC = white blood cell.

## 3 Discussion

We report a case of acute pancreatitis after the first dose of the Pfizer/BioNTech COVID-19 mRNA vaccine. Although there are only a few case reports regarding the development of acute pancreatitis after vaccination against COVID-19, it is extremely important to recognize that acute pancreatitis can develop shortly after COVID-19 vaccination.

The present case developed acute pancreatitis 2 days after the first dose of the COVID-19 vaccine. The patient had no history of alcohol consumption and had no recent changes in her regular oral medication. In addition, hypertriglyceridemia, biliary stones, pancreaticobiliary maljunction, pancreatic tumor, and autoimmune pancreatitis were excluded. Therefore, the COVID-19 vaccine was considered to be involved in the onset of pancreatitis in this case.

Two cases of acute pancreatitis after COVID-19 mRNA vaccine have been reported.^[6,7]^ Both cases were women who had developed pancreatitis within a few days after the first dose of the Pfizer/BioNTech COVID-19 mRNA vaccine. Both cases were mild, and no case of pancreatitis with extensive inflammation, as in this case, has been reported yet. Recently, a case of acute pancreatitis after the second dose of Pfizer/BioNTech COVID-19 mRNA vaccine was also reported.^[8]^ However, this patient had consumed alcohol just before the onset of pancreatitis. Thus, we wonder whether the alcohol consumption rather than administration of COVID-19 mRNA vaccine is the event associated with the development of acute pancreatitis in the report of Walter et al. Although the severity is unknown, the vaccine analysis print based on all spontaneous reports received for the Pfizer/BioNTech COVID-19 mRNA vaccine between December 9, 2020 and September 22, 2021 contains 330,983 reports of side effects, including 1 case of obstructive pancreatitis, 12 cases of pancreatitis, 12 cases of acute pancreatitis, and 1 case of necrotizing pancreatitis.^[9]^ Thus, it is of great importance to recognize that acute pancreatitis can occur after COVID-19 vaccination.

Although the mechanism of vaccine-induced pancreatitis remains unclear, a compelling hypothesis of molecular mimicry (molecular mimicry theory) has recently been proposed.^[10–12]^ According to this theory, cross-reactivity due to the similarity between amino acid sequences of viral and self-antigens leads to tissue damage by the cytotoxic antibodies.^[13,14]^ In support of this hypothesis, antibodies against SARS-CoV-2 spike protein have been reported to be cross-reactive to many human tissue antigens.^[15]^ In addition, Anand et al reported that SARS-CoV-2 evolved its own S1/S2 cleavage site and consequently resembled an identical 8-mer FURIN cleavage peptide on the human epithelial sodium channel α subunit. FURIN is expressed in multiple cell types, including in the intestine, pancreas, and lungs, along with angiotensin converting enzyme 2 and epithelial sodium channel α subunit.^[16]^ Based on these reports, we assumed that COVID-19 mRNA vaccination resulted in an autoimmune response based on molecular mimicry, leading to pancreatic injury. Additionally, given that our patient developed pancreatitis 2 days after vaccination, memory T cells had been sensitized to antigens similar to SARS-CoV-2 before vaccination, because post-vaccination antibody production generally requires several weeks.^[17]^ In addition to pancreatitis, infrequent side effects including myocarditis and Guillain-Barré syndrome that developed after COVID-19 mRNA vaccination have also been reported, and the involvement of molecular mimicry theory has been proposed as a mechanism for their development.^[18,19]^

In conclusion, we report a case of acute pancreatitis that developed soon after the first dose of COVID-19 vaccination. Although it is difficult to conclude that this case developed acute pancreatitis as a side effect of the COVID-19 mRNA vaccine, it is of great importance to diagnose acute pancreatitis when severe abdominal pain occurs soon after COVID-19 mRNA vaccination.

## Author contributions

**Conceptualization:** Takashi Kobayashi, Kazunari Murakami.

**Data curation:** Sotaro Ozaka.

**Formal analysis:** Sotaro Ozaka.

**Supervision:** Kazunari Murakami.

**Writing – original draft:** Sotaro Ozaka.

**Writing – review & editing:** Takamoto Kodera, Shimpei Ariki, Takashi Kobayashi, Kazunari Murakami.
